# Supplementary material for: Single-Cell Sequencing: An Emerging Tool for Biomarker Development in Nuclear Emergencies and Radiation Oncology
Source: Cancers (Basel). 2025 May 28;17(11):1801. doi: 10.3390/cancers17111801 (PMC12153836; doi:10.3390/cancers17111801)
Supplement: Supplementary file 1 [file cancers-17-01801-s001.zip › cancers-3558158-supplementary.pdf]

Supplementary Table S1 Comprehensive Summary of Radiation Biomarker Candidates Identified in the Past Decade.

Biomarker(s)	Sample Type	Radiation Type	Dose	Time	Specificity	Sensitivity	Experiment Model	Validation Methods	Study Method	Reference
**PTC transcriptomic signature[~]**	Thyroid tissue	^{131}I, post-Chernobyl radioiodine fallout	NA	NA	N/A	N/A	Chernobyl tissue bank	qRT-PCR	microarray	[51]
**BRAF mutation, RET/PTC1 rearrangement, and low proliferation index**	PTC tissue	Chernobyl radioactive fallout	NA	18 - 22 yrs	N/A	N/A	Retrospective cohort study	IHC, FISH, qRT-PCR	Pyrosequencing	[46]
**26 metabolite signals[+]**	Plasma, urine	^{137}cesium chloride drinking water	0 - 0.004 Gy	9 mos	N/A	N/A	Male and female Sprague-Dawley rats	N/A	Blood cell counts, Biochemical panel, LC-MS analysis	[84]
**miRNA-150**	Serum	^{137}Cs source	0 - 8 Gy	24, 48 hrs	N/A	N/A	CBA/J and C57BL/6 male mouse model	N/A	miRNA profiling	[67]
**CCNG1, PHPT1**	Peripheral blood	X-ray	0.5 - 4 Gy, 0.005 - 0.1 Gy	2, 24 hrs	N/A	N/A	Healthy human donors	N/A	qRT-PCR	[52]
**HLA-genetic predisposition**	Human blood	Chornobyl accident	0.75 - 2 Gy	NA	N/A	N/A	Hematological and cardiovascular disease patients	N/A	Molecular-genetic examination	[47]
**KDR, CEACAM8, OSM transcripts, CD4+ cells, CD4+/CD8+ ratio**	Peripheral blood	^{60}Co γ-ray	1 - 11.2 Gy	1 - 3 yrs	N/A	N/A	Accidentally exposed people	qRT-PCR	Whole genome gene expression microarray assay, flow cytometry, agarose gel electrophoresis	[53]
**miR-21**	Serum	X-ray	50 Gy	5 wks	75%	80%	Breast cancer patient cohort	N/A	qRT-PCR	[68]
**absolute neutrophil count, monocyte count, CRP**	Peripheral blood	^{60}Co γ-ray	2.5 - 15 Gy	0 - 7 d	N/A	N/A	Adult male baboons	N/A	Logistic regression analysis, blood cell count	[72]

**CD11b+CD13+, CD29+CD13+, cell adhesion and migration**	Whole blood, PBMC	γ-ray	0 - 6 Gy	6, 12, 24 hrs	N/A	N/A	Male students (19 - 24 yrs), male Sprague Dawley rats	N/A	Enzyme-linked immunoassay, transwell chamber assay	[73]
**CLIP2**	PTC tissue	post-Chernobyl radioiodine fallout	mean: 0.59 vs 1.2 Gy	mean: 14 yrs vs 10 yrs	82.4% for the Genrisk-T cohort. 57.1% for Genrisk-T-PLUS cohort.	72.4 - 75.0%	Retrospective cohort study	IHC, qRT-PCR, logistic regression analysis	mRNA microarray	[54,55]
**miRNA signatures***	Serum	^{137}Cs source	0 - 8 Gy	24 hrs - 1 mos	See comments	N/A	C57BL/6J male mice, HuCD34+ humanized NSG mice	qRT-PCR	miRNA profiling	[69]
**PHA**	Peripheral blood slides	Y-12 accident	2.98 - 4.61 Gy, 0.29 - 0.86 Gy	9 hrs - 16 yrs	N/A	N/A	Y-12 retrospective cohort study	N/A	Blood smear staining	[87]
**Agpat9, Plau, Prf1, S100a8 genes**	Thyroid tissue	^{131}I	0 -17 Gy	24 hrs	N/A	N/A	Adult BALB/c nude mice	N/A	RNA microarray	[56]
**Gene signatures%**	Peripheral blood, PBMC	X-ray	0 - 1 Gy	8, 24hrs	N/A	N/A	Healthy blood donor	qRT-PCR	Microarray	[57]
**NF-κB1, NF-κB2, Rel genes**	Peripheral blood, bone marrow	post-Chernobyl	NA	22 - 23 yrs	N/A	N/A	Ukrainian Patients with Leukemia and Lymphoma	N/A	qRT-PCR	[60]
**lectin-erythrocyte interactions**	Peripheral blood erythrocytes	Chernobyl radioactive fallout	0 - 2+ Gy	4 - 10 yrs	N/A	N/A	Male cohort	N/A	Modified microplate lectinoassay	[89]
**lipid metabolism (postnatal), steroid hormone metabolism (adult)**	Plasma, serum	^{137}cesium chloride drinking water	adult: 4.4 ± 1.3 mGy, postnatal: 0.30 ± 0.05 mGy, in utero: 0.96 ± 0.15 mGy.	0 - 12 mos	N/A	N/A	Sprague–Dawley rats adult, postnatal and in utero models	N/A	Spectrophotometric system, ELISA	[85]
**PHA**	Peripheral blood slides	Y-12 accident; ^{60}Co γ-ray	0.29 - 4.61 Gy; 1 - 8.5 Gy	12 hrs - 16 yrs; 5	N/A	N/A	Y-12 retrospective cohort study; NHP	N/A	Blood smear staining	[88]

				hrs - 60 d						
**5-miRNA composite signature[&]**	Serum	X-ray	0 - 7.2 Gy	-7 d, 24 hrs	Model: 86.7%, Classifier: 95%	Model: 75.0%, Classifier: 95%	NHP	qRT-PCR	miRNA profiling	[71]
**Urinary metabolic signatures[^]**	Urine	NA	0 - 8 Gy	5 - 48 hrs	N/A	N/A	Male Wistar rats	N/A	GC-MS analysis, LC-MS/MS	[86]
**telomere length, chronic viral infection**	Lymphocytes	Chernobyl radioactive fallout	602.67 ± 114.19 mSv	30+ yrs	N/A	N/A	Chornobyl cleanup male workers	N/A	FISH, flow cytometry	[74]
**TCR-CD4+, γ-H2AX+ and CyclinD1+ cell counts**	Human blood	γ-ray and incorporation of transuranium elements	0.1 - 113.35 mSv (average: 26.06 mSv)	7 - 14 d	N/A	N/A	Shift radiation workers at the Shelter object and inside Chornobyl exclusion zone	qRT-PCR	Flow cytometry approach	[90]
**CNV**	Peripheral blood	^{137}Cs	≤ 0.2 Gy	NA	N/A	N/A	Goiânia accident retrospect families	N/A	Chromosomal microarray analysis	[48]
**Cellular immunity, gene expression, telomere length, intracellular protein parameters[‼]**	NA	Post-Chernobyl radioiodine fallout	0.10 - 3,500 mSv	NA	N/A	N/A	Chornobyl accident male clean-up workers	qRT-PCR	flow FISH, flow cytometry	[58]
**SAA1**	Serum	^{60}Co γ-ray	1 - 12 Gy	0.25 - 7 d	91% in mice. 88% in patients	100% in mice, 100% in patients	C57BL/6J female mice	qRT-PCR	ELISA	[45]
**Radiation-responsive “signature” genes[$]**	Human blood	γ-ray or X-ray	0.1 - 8 Gy	24 hrs	N/A	N/A	NCBI GEO database	qRT-PCR	Meta-analysis	[61]
**Glutathione transferase, glutathione peroxidase**	Blood	^{137}Cs contamination	185 - 8806 Bq	10 - 17 yrs	N/A	N/A	Children residents of radioactive-contaminated territories	N/A	Pneumotachographic method, radiation detector scan	[75]
**Keratins K1 and K10**	Serum	Uranium	0–874.3 mSv (mean: 53.5	7.4 yrs (exposure)	80% (CYFRA21-1)	80% (CYFRA 21-1)	Wismut uranium miners cohort	MALDI-TOF-MS	Two-dimensional gel electrophoresis	[76]

			± 101.7 mSv)							
**AMY1A, FLT3L, MCP1**	Plasma	X-ray or γ-ray	0 - 10 Gy	24 hrs - 14 d	94.00%	92.10%	NHP	Immunoassay, targeted quantitative mass spectroscopy assay	Tandem mass spectrometry	[77]
**CLIP2-PPIL3 co-expression**	TPC-1 cells	NA	NA	NA	N/A	N/A	in vitro	qRT-PCR, IHC, western blot	Microarray	[62]
**ERCC1, ESCO2**	DNA repair genes	γ-ray	0.1 - 5 Gy	NA	N/A	N/A	GEO datasets	N/A	In silico method	[63]
**A2m, CHGA, GPX3**	Plasma	^{60}Co γ-ray	0 – 5 Gy	1 – 7 d	N/A	N/A	Sprague-Dawley rats	ELISA	LC-MS/MS	[78]
**MD of autosomal SNPs**	Peripheral blood	Goiânia ^{137}Cs accident	0.2 – 0.5 Gy	NA	N/A	N/A	Case-control observational study	N/A	Chromosomal microarray analysis, SNP genotypes	[49]
**Gene expression and cellular markers**[@]	Whole blood	γ-ray	2.16 – 84.16 mSv	7 – 14 d	N/A	N/A	Shift radiation workers at the Shelter object and inside the Chornobyl exclusion zone	qRT-PCR	Flow cytometry	[59]
**γ-H2AX mean fluorescence intensity**	Peripheral blood	^{137}Cs source	0 – 2.7 Gy	0 – 7 d	N/A	N/A	C57BL/6 male mice	N/A	Imaging flow cytometry	[79]
**30+ proteomic biomarkers**	Serum	Fukushima Daiichi nuclear power plant accident	0.001 – 0.64 Gy	lifetime	N/A	N/A	Wild field mice (Apodemus speciosus)	N/A	Aptamer-based SOMA-scan assay	[80]
**15 mRNAs**[#]	Peripheral blood	Radium-223 dichloride	4 MBq/cycle	6 mos	N/A	N/A	Advanced-stage prostate cancer patients	qRT-PCR	Whole genome mRNA seq and small RNA seq	[64]
**BPIFA2**	Serum	^{60}Co γ-ray	0.5 – 10 Gy	1 – 12 hrs	High (only detected in the parotid gland)	High (Ionizing radiation)	C57BL/6J female mice	ELISA, Western Blot	Proteomics	[44]

**PPHA**	Peripheral blood slides	X-ray or ^{60}Co γ-ray	0 – 8.5 Gy	3 yrs	N/A	N/A	Rhesus macaques	N/A	Peripheral blood smears	[91]
**131I exposure novel biomarkers!**	Thyroid tissue, plasma	131I	0.1, 0.07 Gy	3 – 9 mos	N/A	N/A	Sprague Dawley rats	N/A	Microarray, LC-MS/MS analysis	[65]
**GRB7, B2M, PMAIP1**	MIAPaCa-2 cells	Proton or X-ray	2, 8 Gy	3, 12 hrs; 4 hrs (reirradiation)	N/A	N/A	GEO database NCBI	N/A	Gene expression microarray, Stemchecker, Omicsbean	[66]
**C-3SFBP, C-7IUVU**	Dermal fibroblast cells	NA	0 - 4 Gy	2 – 3 wks	N/A	N/A	Healthy human (M & F) fibroblast cell donors	N/A	Microarray, SF2, dynamic programming	[50]
**BRAF/NRAS mutation, PD-L1, PD-1, P16INK4A, Ki-67**	PTC tissue	^{131}I, post Chernobyl radioiodine fallout	NA	NA	N/A	N/A	Chernobyl tissue bank	N/A	IHC, histopathological examination	[81]
**CXCL10, FDXR**	Peripheral blood, PBMC	X-ray or ^{137}Cs-ray source	1, 3 or 5 Gy (1 Gy/min); 3.25 Gy/min	24, 48, 72 hrs; 24 hrs	N/A	N/A	C57BL/6 female mice, human PBMC donors	qRT-PCR, digital real-time PCR	Blood cell count, microarray	[43]
**miRNA signatures****	Whole blood, plasma	X-ray	0, 20, 40, 80 Gy (2.5 Gy/min)	8 – 11 wks	N/A	N/A	C57BL/6J mice	qRT-PCR	miRNA profiling, complete blood counts, CRP quantitative ELISA	[70]
**Serum sSelectin-L**	Blood, serum	γ-ray or X-ray	0, 0.5, 1, 2, 4, 6.5, 10 Gy (0.011 Gy-1); 63/70 Gy	2 – 4 wks; 1 -4 d	N/A	N/A	C57BL/6J male mice, nasopharyngeal carcinoma patients	N/A	ELISA	[82]
**BAX, DDB2**	Blood	X-ray	0 – 5 Gy	48 hrs	88.89% (Humans)	100.00% (Humans)	Healthy humans, rhesus macaques	ELISA	ELISA	[83]

~ serpine peptidase inhibitor clade E (SERPINE1), dual specificity phosphatase 1 (DUSP1), tribbles homologue 1 (TRIB1), S100 calcium-binding protein A10 (S100A10), annexin A1 (ANXA1), guanine nucleotide-binding protein G(olf) subunit alpha (GNAL) and retinol dehydrogenase 12 (RDH12).
+ 26 metabolite signals include 2 urinary, 4 plasma non-polar, and 19 plasma polar metabolites, which are not specifically listed.

* e.g., miR-126-3p, miR-150, miR-342-3p, miR-151-3p, miR-139-3p, and miR-142.
% DDB2, POLH, MDM2, RPS27L, FDXR, CCNG1, TRIAP1, SESN1, FBXO22, PPM1D, ANKRA2, CDKN1A, TRIM22, and BBC3.
& a compound classifier, including the radiation biomarker miRNAs (miR-133b, miR-215, and miR-375), the survival indicators (miR-30a and miR-126), and normalizer miRNAs (miR-142 and miR-320a).
^ Oxalic acid, Phosphoric acid, Oxoglutaric acid, Citric acid, ippuric acid, Trehalose, L-Aspartic acid, Citrulline.
!! BCL2, SERPINB9, CDKN2A, STAT3, TP53, IL1B, MCF2L, TNF genes, γ-H2AX and Cyclin D1 expression, telomere shortening.
$ ANKRA2, DRAM1, RPS27L, ANXA4, GADD45A, SESN1, ARHGEF3, GDF15, SLC4A11, ASCC3, IL21R, SLC7A6, ASTN2, LIG1, TNFRSF10B, BBC3, MAMDC4, TRIAP1, HIST1H2BD, MAP4K4, UROD, CDKN1A, PCNA, VWCE, DDB2, PHPT1, WIG1, EI24, PLK3, XPC, FBXO22, PPM1D, ZNF337, FDXR, PTP4A1, ZNF541, REV3L.
@ phosphorylated gamma-H2AX and DDB2, TP53, BRCA1, CCND1, BIRC5, TERT, VEGFA, BAX, NFKB1 genes, and telomeres length.
# CXCL5, DMTN, FBXO7, GNG11, MAP3K7CL, SH3BGRL2, TMEM56-RWDD3, TUBB2A, RNF11, PF4, BZW1, PDZK1IP1, PPBP, RAB27B, CXCL8.
! (PTH), age-dependent dose response (CA1, FTL1, PVALB (youngsters) and HSPB6 (adults)), thyroid function (Vegfb (adults)).
** WBI: miR-150-5p, miR-342-3p, miR-146a-5p; Inflammation: miR-18a-5p, miR-148b-3p, miR-532-5p; skin disorders: miR-139-5p, miR-195-5p.

PTC: Papillary thyroid cancer. FISH: fluorescence in situ hybridization. BRAF: v-raf murine sarcoma viral oncogene homolog B1. RET: Ret Proto-Oncogene. CCNG1: Cyclin1. PHPT1: phosphohistidine phosphatase 1, HLA: human leukocyte antigen. KDR: kinase insert domain receptor. EACAM8: carcinoembryonic antigen-related cell adhesion molecule 8. OSM: oncostatin M. CRP: C-reactive protein. IHC: immunohistochemistry. NF-κB: Nuclear factor kappa B. PHA: pseudo-Pelger Huët anomaly. NHP: nonhuman primate. GC-MS: Gas chromatography-mass spectrometry. LC-MC/MC: Liquid Chromatography Triple Quadrupole Mass Spectrometry. TCR: T-cell receptor. CNV: copy number variants. MALDI-TOF-MS: Matrix-assisted laser-desorption ionization time-of-flight mass spectrometry. AMY1A: Salivary Alpha Amylase. FLT3L: Fms-related tyrosine kinase 3 ligand. MCP1: Monocyte Chemotactic Protein 1. TPC: CLIP2-perturbed thyroid cancer cell line. PPIL3: Peptidylprolyl Isomerase Like 3. ERCC1: ERCC excision repair 1. ESCO1: establishment of sister chromatid cohesion N-acetyltransferase 1. A2m: alpha-2-macroglobulin. CHGA: chromogranin-A. GPX3: glutathione peroxidase 3. MD: mendelian deviations. SNPs: single-nucleotide polymorphisms. ELISA: enzyme-linked immunosorbent assay. PPHA: pseudo Pelger-Huët anomalies. C-3SFBP: located within an intron in the gene MCC. C-7IUVU: associated with the SLC1A6 gene. GRB7: growth factor receptor-bound protein 7. MIAPaCa-2: pancreatic cancer cells. CXCL10: C-X-C motif chemokine ligand 10. FDXR: ferredoxin reductase.
